# IL-1 Family Members in Cancer; Two Sides to Every Story

**DOI:** 10.3389/fimmu.2019.01197

**Published:** 2019-06-07

**Authors:** Kevin J. Baker, Aileen Houston, Elizabeth Brint

**Affiliations:** ^1^Department of Pathology, University College Cork, Cork, Ireland; ^2^Department of Medicine, University College Cork, Cork, Ireland; ^3^APC Microbiome Ireland, University College Cork, Cork, Ireland; ^4^CancerResearch@UCC, University College Cork, Cork, Ireland

**Keywords:** interleukin-1 (IL-1), inflammation, cancer, IL-18, IL-33, IL-36 family interleukins

## Abstract

The IL-1 family of cytokines currently comprises of seven ligands with pro-inflammatory activity (IL-1α and IL-1β, IL-18, IL-33, IL-36α, IL-36β, IL-36γ) as well as two ligands with anti-inflammatory activity (IL-37, IL-38). These cytokines are known to play a key role in modulating both the innate and adaptive immunes response, with dysregulation linked to a variety of autoimmune and inflammatory diseases. Given the increasing appreciation of the link between inflammation and cancer, the role of several members of this family in the pathogenesis of cancer has been extensively investigated. In this review, we highlight both the pro- and anti-tumorigenic effects identified for almost all members of this family, and explore potential underlying mechanisms accounting for these divergent effects. Such dual functions need to be carefully assessed when developing therapeutic intervention strategies targeting these cytokines in cancer.

## Interleukin-1 Superfamily

### Introduction

The importance of inflammation in cancer is now well established (1–3). In some cancers, the inflammatory conditions precede the development of malignancy, e.g., chronic bronchitis is a major risk factor for lung cancer. Alternatively, aberrant signaling due to oncogenic mutations in tumors can result in a chronic inflammatory state developing both proximal to, and within, the tumor. This chronic inflammation acts to inhibit the anti-tumorigenic immune response, normally mediated by cells such as M1 macrophages, NK cells and CD8^+^ T cells. Tumor cells themselves can also directly induce an immunosuppressive microenvironment through recruitment and activation of specific immune cell subtypes such as myeloid derived suppressor cells (MDSC), M2 macrophages and T regulatory cells, further promoting tumorigenesis (2, 4, 5). Understanding the complexity of immunomodulation by tumors is important for the development of effective immunotherapies. Cytokines, such as Interleukin-1 (IL-1) are central mediators of the interactions between cells in the inflammatory tumor microenvironment. The IL-1 family now includes seven ligands with pro-inflammatory activity (IL-1α and IL-1β, IL-18, IL-33, IL-36α, IL-36β, IL-36γ) as well as anti-inflammatory cytokines (IL-37, IL-38). Several members of this family, such as IL-1β and IL-18, have been extensively investigated in cancer with both pro- and anti-tumorigenic functions ascribed to these cytokines. In contrast, far less is currently understood concerning the role of more recently identified members of this family in cancer such as IL-33 and IL-37, although such data that is available also indicates that these may have both pro- and anti- tumorigenic effects. In this review, we will explore the dual functions of these cytokines in the tumorigenic process.

## Interleukin-1 (IL-1)

As IL-1 was the original defining member of its family, its physiological function and indeed, pathophysiological function, has been extensively studied and reviewed (6). The triumvirate of cytokines grouped under the heading of IL-1 includes two of the activator cytokines IL-1α and IL-1β, as well as an inhibitory cytokine, the IL-1 receptor antagonist. IL-1α and IL-1β bind the same receptor, the type 1 IL-1 receptor (IL-1R), recruiting both the IL-1R accessory protein and the adaptor protein MyD88 to the receptor complex, resulting in activation of the downstream signaling cascade and ultimately in the activation of a myriad of immune and inflammatory genes (7). Whilst these cytokines activate through the same pathway, they are separately encoded proteins with a low level of sequence homology and well characterized divergent biological processes, cellular localization and mechanisms of activation. Both IL-1α and IL-1β exist as pro-forms and cleaved forms, but whereas both forms of IL-1α are biologically active, only the cleaved form of IL-1β acts as a pyrogen. IL-1α is grouped in a category of dual function cytokines (with IL-33 and IL-37), as it is located both within the nucleus of the cell where it plays a role in transcription (8), and also as a functional membrane bound cytokine. In addition, IL-1α is released from necrotic cells allowing it to function as an “alarmin.” In contrast, the processing and bioavailability of IL-1β is very tightly controlled. We now know that IL-1β requires a “two-signal” process to become activated (9), with the initial priming signal triggering transcription of the gene and the second signal, resulting in inflammasome activation, allowing caspase-1 mediated cleavage and activation of IL-1β. It is therefore, not surprising that these two cytokines, so often grouped together, have been shown to have different physiological and biological effects in many human diseases and indeed, with relevance to this review, in different cancer types (10). Also, as with so many of this family of cytokines, divergent pro- and anti- tumorigenic effects have been reported for both. Here we will detail some of these alternate functions ascribed to both cytokines.

## IL-1α

### Tumor-Promoting Effects of IL-1α

Whilst there is no shortage of data supporting a role for IL-1β in tumorigenesis, there are relatively fewer studies directly examining IL-1α in cancer development and progression. IL-1α expression has been reported to be significantly increased in head and neck squamous carcinoma (HNSCC) patients with distant metastasis as compared to those without metastasis (11). A similar correlation between IL-1α expression and distant liver metastasis has been reported in patients with gastric carcinoma (12). High levels of IL-1α expression in cancers have also been linked to tumor dedifferentiation (13) and lymphangiogenesis (14). Associations between single nucleotide polymorphisms in the IL-1α gene and cancer risk have been identified (15) with carriers of the rs17561 missense mutation showing reduced susceptibility to clear cell ovarian cancer.

Both *in vitro* and *in vivo* evidence have supported this clinical data. Interestingly, both forms of IL-1α have been shown to have potential tumor promoting effects (16) as the pro-piece, nuclear form of IL-1 has been shown to facilitate the growth of acute T-lymphocytic leukemia cells through the activation of NF-κB and SP1. Stable overexpression of IL-1α in the normally non-metastatic IL-1α negative pancreatic carcinoma cell line MIaPaCa-2 enhanced NF-κB production and cellular invasion *in vitro* (17), and resulted in liver metastasis following orthotopic injection in murine models. Moreover, co-culture of pancreatic ductal adenocarcinoma cells with cancer-associated fibroblasts (CAFs) resulted in marked upregulation of multiple inflammatory factors including IL-1α. Inhibition of IL-1α alone reduced the crosstalk-induced production of these inflammatory factors (18), indicating a key role for IL-1α in the formation and maintenance of the inflammatory tumor environment. Pancreatic cancer cell-derived IL-1α was similarly shown to promote hepatocyte growth factor secretion by fibroblasts (19), promoting pancreatic cancer invasion, proliferation and angiogenesis. IL-1α expression was also essential for tumor development following implantation of the 3-MCA-induced fibrosarcoma cell line (20), with findings from this study highlighting an important role for IL-1α in controlling immune-surveillance of the developing tumor. A similar role for Il-1α was recently demonstrated in breast cancer (21). Breast tumor-derived IL-1α, acting on tumor-infiltrating myeloid cells, induced the expression of thymic stromal lymphopoietin (TSLP), which was not only a critical tumor survival factor, but is also required for the metastatic spread of the tumor cells. These studies define a novel IL-1α-TSLP-mediated crosstalk between tumor-infiltrating myeloid cells and tumor cells in the control of metastatic breast cancer. Finally, recent elegant work examining the relationship between HER2 expression in breast cancer, inflammation and expansion of cancer stem cells (CSCs) highlighted an essential role for IL-1α in this process (22). These authors demonstrate that HER2 expression in the tumor drives a positive feed-forward activation loop, inducing the expression of IL-1α and IL-6. This, in turn, increases activation of NF-κB and STAT3, allowing for the generation and maintenance of CSCs. IL-1α- deficient mice show delayed MMTV-Her2-induced tumorigenesis, reduced inflammation and reduced numbers of CSCs. Moreover, pharmacologic blockade of IL-1α signaling reduced the population of CSCs in the tumors, and improved chemotherapeutic efficacy (22).

Given these tumor-promoting functions for IL-1α, studies have investigated whether it represents a potential therapeutic target for cancer therapy. MABp1 (XBiotech Inc.) is a naturally occurring human IL-1α neutralizing antibody which has shown promising outcomes in several cancer types (23, 24). Also, a recent randomized phase III trial by Kurzrock et al. showed patients with advanced colorectal cancer and lower levels of circulating IL-1Ra are more responsive to treatment with bermekimab, an antibody targeting IL-1α. Levels of IL-1Ra were shown to be reflective of the potentiated response from antibody administration (25). Further work in this field will identify whether mechanisms for neutralizing IL-1α can be optimized and made viable for cancer therapy.

### Tumor-Suppressive Effects of IL-1α

In contrast to the above findings, studies have shown that, particularly transient, overexpression of IL-1α may have anti-tumorigenic effects. Both fibrosarcoma and lymphoma cells were induced to express IL-1α in a transient manner. Of note, in this model IL-1α was expressed in the cytosol and not excreted. The IL-1α-expressing tumor cells were seen to predominantly not result in tumor development. Furthermore, if tumors did occur, they subsequently regressed. Tumor regression was observed to be mainly mediated by CD8^+^ T cells, with some involvement of NK cells and macrophages (26, 27). Moreover, stimulation of multiple cancer cell lines, including MCF-7 breast cancer, A375 melanoma, prostate stem cells and murine primary mammary cells, with IL-1α inhibited cell proliferation by causing G0–G1 cell cycle arrest. (28–30). More recently, a study investigating the role of the IL-1R in tumorigenesis indicated that IL-1R signaling could suppress mammary tumor cell proliferation in the MMTV-PyMT breast cancer mouse model (31). This phenotype was shown to be IL-1α dependant, indicating that IL-1α-IL1R signaling may be tumor-suppressive in PyMT-driven breast cancer.

## IL-1β

### Tumor-Promoting Effects of IL-1β

Evidence supporting a pro-tumorigenic role for IL-1β across all cancer types has been accumulating over many years and has been extensively reviewed in recent times (30). Solid tumors in which IL-1β has been shown to be upregulated include breast, colon, lung, head and neck cancers, and melanomas, and patients with IL-1β producing tumors generally exhibit a poorer prognosis (32). Multiple strands of both *in vitro* and *in vivo* evidence point toward a tumor-promoting function for IL-1β. Fibrosarcoma cells, genetically modified to constitutively excrete mature IL-1β, show increased growth, invasiveness and angiogenesis (33). Indeed, clear associations have been demonstrated between IL-1β and angiogenesis in multiple tumor types (34, 35). In a transgenic model of Myc-dependent carcinogenesis, IL-1β has been characterized as the principal effector molecule in the onset of angiogenesis (36). Mechanistically, this was seen to be mediated via matrix metalloproteinase (MMP) -induced sequestration of extracellular matrix–associated vascular endothelial growth factor (VEGF), followed by its ligation to VEGFR2 on endothelial cells. Moreover, in a model of melanoma, myeloid cells was shown to produce IL-1β (37), which subsequently activated endothelial cells to produce VEGF and other proangiogenic factors, modulating the inflammatory microenvironment of the tumor, allowing for enhanced angiogenesis and tumor progression.

As could be predicted based on its ability to enhance angiogenesis, IL-1β has also been shown, in multiple models across multiple cancer types, to enhance tumor cell metastasis. In both murine and human breast cancer models, tumor progression was associated with the activation of inflammasome components and subsequent elevated levels of IL-1β at primary and metastatic sites. Mice deficient for inflammasome components exhibited significantly reduced tumor growth and lung metastasis (38). Similar findings have been shown for the NLRP3 inflammasome in lymphangiogenesis and metastasis (39). Macrophage-dependent lymphangiogenesis was triggered upon inflammasome activation and required IL-1β production. NLRP3 expression in tumor-infiltrating macrophages also correlated with survival, lymph node invasion, and metastasis of mammary carcinoma patients (39). The role of IL-1β in driving expression and production of downstream pro-tumorigenic cytokines appears to play a key role in this process. IL-1β induces IL-6 production in breast cancer cells, as well as additional cytokines and growth factors including TGF-β, TNF-α, and EGF. Indeed, these cytokines and growth factors seem to potentiate the effect of IL-1β on IL-6 expression. In breast cancer cells, epithelial-to-mesenchymal (EMT) and stem-cell-like phenotypes were attenuated by either anti-IL-6 or anti-IL-1β antibody treatment (40). A similar causal link between IL-1β and IL-22 has recently been established (41). IL-22 is now emerging as a cytokine with potent tumor-promoting properties. It has recently been shown that IL-22 production in humans is dependent on activation of the NLRP3 inflammasome with the subsequent release of IL-1β from both myeloid and T cells. The IL-1 receptor antagonist Anakinra abrogates IL-22 production and reduces tumor growth in a murine breast cancer model (40).

Based on these, and many other (42), findings, IL-1β is now viewed as an attractive target for pharmacological intervention. Several agents are available including IL-1Ra (Anakinra) which inhibits both IL-1α and IL-1β signaling, neutralizing antibodies to IL-1β (Canakinumab), as well as inflammasome inhibitors. It has recently been shown that administration of inhibitory antibodies targeting IL-1β results in the augmentation of anti-tumor cell immunity in a murine model of breast cancer. A synergistic effect of anti-IL-1β and anti-PD-1 treatment with antibodies was capable of altering TME cell populations in favor of an anti-tumor phenotype (43). In clinical trials, Anakinra has been shown to inhibit IL-6 production and enhance the progress-free survival of patients with indolent myeloma (44). Moreover, a recent comprehensive study convincingly demonstrated that Anakinra may also be beneficial in the treatment of breast cancer (43). In this study, production of IL-1β in primary breast cancer tumors was linked with advanced disease. IL-1β was shown to originate from tumor-infiltrating CD11c^+^ myeloid cells with production triggered by cancer cell-derived TGFβ. Administration of anakinra prevented breast cancer progression in humanized mouse model. Moreover, a pilot clinical trial wherein 11 patients with Her2^−^ metastatic breast cancer were treated with daily subcutaneous Anakinra for a median duration of 4 months, in combination with one of the standard chemotherapeutics, demonstrated that high risk Her2^−^ breast cancer patients would benefit from reducing levels of IL-1β and treatment with anakinra (45, 46). Excitingly, a recent phase three clinical trial (CANTOS) (45) demonstrated that an inhibitory antibody targeting IL-1β (canakinumab) could significantly reduce the incidence and mortality of lung cancer. Although this trial involving 10,500 patients was not initially designed to study lung cancer as an end-point, analysis revealed that there was a 56% reduction in incident lung cancer in the canukinumab-treated group as compared to the placebo group. As these patients were extensively screened prior to recruitment, the indication is that inhibition of IL-1β reduced the progression, invasiveness and metastatic spread of early stage lung cancers that were undiagnosed at time of recruitment. These data emphasize the importance of this cytokine in chronic inflammation in lung tumorigenesis, and its validity as a therapeutic target.

### Tumor Inhibiting Effects of IL-1β

**Figure 1:**
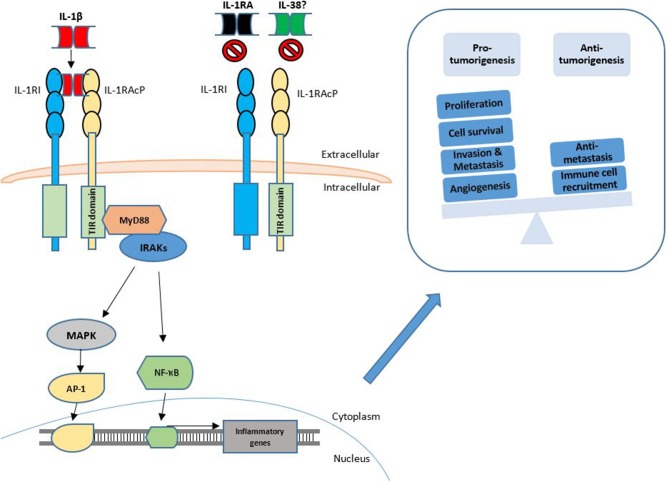
Functions of IL-1β signaling in cancer. The IL-1 signaling pathway is activated through the binding of IL-1α or IL-1β to the IL-1RI. This is followed by dimerization of IL-1R1 with IL-1RAcP. This interaction may be competitively inhibited by the IL-1RA and IL-38. Agonist binding is followed by engagement of further signaling intermediates with subsequent activation of transcription factors such as NF-κB and AP-1. Transcription of inflammatory genes is upregulated which may then result in pro-tumorigenic or anti-tumorigenic phenotypes.

The divergent and conflicting role of the IL-1 cytokines is particularly apparent when investigating IL-1β. The majority of these studies highlight the ability of IL-1β to induce both Th1 and Th17 responses and thus to induce anti-tumorigenic effects. Exogenous injection of IL-1β resulted in tumor regression, as long as the tumors were of a sufficient size, and the mice were not deficient in T cells (47). Haabeth and colleagues also demonstrated that IL-1β drives activation of tumor specific Th1 responses, thus protecting against B cell myeloma and lymphoma (48, 49). Contrasting tumor-inhibiting effects have also been ascribed to IL-1β in the case of colorectal cancer (CRC). Inhibition of the NLRP3 inflammasome was seen to reduce tumor burden in the murine model of colitis-associated cancer, with the increased tumor burden correlating with attenuated levels of IL-1β and IL-18 at the tumor site (50). An insight into the underlying mechanism concerning the dichotomous role of IL-1β in CRC was recently provided. Dmitrieva-Posocco and colleagues examined the impact of IL-1 signaling in different cell types within the CRC microenvironment (51). Analysis of epithelial cell specific IL-1R1 deletion revealed a decrease in CRC tumor multiplicity, slower proliferation of early tumor seeds, and decreased activation of NF-κB. T cell specific ablation of IL-1R1 similarly decreased tumor-elicited inflammation dependent on IL-17 and IL-22, thereby reducing CRC progression. In contrast, IL-1 signaling in myeloid, particularly neutrophil populations, was shown to be potently anti-tumorigenic due to their role in controlling the local microbiota populations. These authors demonstrate that IL-1R signaling in myeloid cells is responsible for keeping specific species/genera of tumor infiltrating microbes at bay, thus preventing local, tumor-specific dysbiosis and excessive amounts of pro-tumorigenic inflammatory cytokines. Interesting interactions between primary tumors and distant metastatic sites in breast cancer, involving IL-1β, have also recently been elegantly described. These authors demonstrate that certain primary tumors elicit a systemic inflammatory response involving IL-1β-expressing innate immune cells that then infiltrate distant metastatic microenvironments. At the metastatic site, IL-1β maintains metastatic cells in a ZEB1-positive differentiation state, preventing them from establishing themselves. In line with these findings (52), the authors further demonstrate that inhibition of the IL-1 receptor relieves the differentiation block and results in metastatic colonization, and that among patients with lymph node-positive breast cancer, high primary tumor IL-1β expression is associated with better overall survival and distant metastasis-free survival. Contrasting this, however, Li et al have provided evidence indicating an opposing role of IL-1β through ZEB1 activation resulting in the promotion of stemness and invasion of colon cancers cells (53). It is clear, therefore, that these dichotomous findings regarding IL-1β need to be carefully considered when developing anti-cancer therapies designed to inhibit IL-1β. The divergent roles of IL-1β in cancer are shown in Figure 1.

## Interleukin-18

Interleukin-18 (IL-18) (also known as IFN-γ-inducing factor) was first identified in 1989 (54) as a factor that enhanced IFN-γ production from anti-CD3-stimulated Th1 cells, especially in the presence of IL-12. Subsequent isolation and characterization of the cytokine determined that its structure was similar to that of IL-1 (55). Similar to IL-1β, IL-18 is synthesized as an inactive 24kDa pro-peptide that is activated by proteolytic cleavage by caspase-1 in the NLRP3 inflammasome, generating the mature, bioactive, 18 kDa molecule. The IL-18 receptor (IL-18R) consists of the inducible component IL-18Rα (IL-1 receptor-related protein [IL-1Rrp]) and the constitutively expressed component IL-18Rβ (IL-1R accessory protein-like [IL-1RAcPL]), and activates a similar pathway to that of IL-1 (55). The activity of IL-18 is suppressed following binding by the high affinity, naturally occurring IL-18 binding protein (IL-18BP), which prevents IL-18 binding to its receptor. IL-18 is expressed by many cell types and plays an important role in both the innate and adaptive immune response (56).

### Tumor-Promoting Effects of IL-18

Numerous studies have investigated the role of IL-18 in cancer, with both pro- and anti-tumorigenic functions identified (56). Some of the first evidence that IL-18 can promote tumor growth came from the observation of elevated expression of IL-18 by tumor cells or elevated levels of IL-18 in the serum of cancer patients, with elevated levels of IL-18 associated with poor prognosis (57–59). These high levels of IL-18 may play a role in angiogenesis, tumor cell migration, invasion and metastasis. Indeed, studies in gastric cancer showed that IL-18 induces the expression of the pro-angiogenic factor, vascular endothelial growth factor (VEGF) (60). VEGF, in turn, can increase the expression and processing of IL-18 (61, 62), with IL-18 increasing cell migration directly through filamentous-actin polymerization and tensin downregulation (61, 62). Consistent with cell migration and angiogenesis being key steps in tumor metastasis, suppression of IL-18 expression in gastric cancer cells resulted in a reduction in both primary tumor growth and metastasis *in vivo*. Moreover, angiogenesis was greatly reduced in tumors lacking IL-18 relative to control tumors (60).

Additional mechanisms proposed for the migratory and metastatic ability of IL-18 involve alterations in expression of adhesion molecules and tight junction proteins (63–65). For instance, administration of IL-18BP significantly suppressed melanoma cell metastasis to the liver by preventing IL-18-induced expression of adhesion molecules such as vascular cell adhesion molecule-1 (VCAM-1) on hepatic sinusoidal endothelial cells (63). Exogenous IL-18 also enhanced the migration of breast cancer cells (65), and this was due to suppression of expression of the tight junction proteins, in particular, claudin-12.

Other tumor-promoting effects associated with IL-18 include evasion of the anti-tumor immune response. Natural Killer (NK) cells, if appropriately activated, play an important role in tumor rejection. However, NK cells can also have immunosuppressive effects. Administration of recombinant IL-18 was shown to up regulate the expression of PD-1 by conventional NK cells, and to convert them into a subset with immunosuppressive characteristics (66, 67). Silencing of IL-18 or blockade of IL-18 activity with IL-18BP, in turn, led to the restoration of NK cell-dependent immune surveillance in tumors (67). IL-18 has also been implicated in the generation of myeloid derived suppressor cells (MDSC). MDSC are potent suppressors of the anti-tumor immune response, with administration of IL-18 shown to increase the population of MDSC in multiple myeloma and in a murine model of melanoma (68, 69). Together, these studies suggest that targeting IL-18 may represent a potential mechanism to overcome tumor-associated immunosuppression.

However, phase 2 clinical trials for metastatic melanoma by GSK utilizing a recombinant human IL-18 (Iboctadekin) demonstrated insufficient clinical benefit (70). It is possible, however, that combining an IL-18 targeting strategy with additional chemotherapeutic reagents could be a better approach in the treatment of patients with cancer. Indeed, a dose-escalation study of recombinant Iboctadekin in combination with rituximab (anti-CD20) was well tolerated and showed increased production of IFN-γ, GM-CSF and chemokines in a pilot study of 5 patients with non-Hodgkin's lymphoma (71).

### Tumor-Inhibiting Effects of IL-18

IL-18, however, has also be shown to activate the anti-tumor immune response, resulting in the suppression of tumor growth and metastasis in multiple tumor types. For instance, administration of recombinant IL-18 has been shown to activate CD4^+^ T cells and/or NK cell immune responses, suppressing the growth and metastasis of melanoma cells *in vivo* (72). Consistent with this, individuals with low NK cell activity have an increased risk of developing cancer (73), with high numbers of intratumoral NK cells often correlated with improved prognosis for cancer patients (74, 75).

These opposing effects described for IL-18 in tumorigenesis could be due to the level of IL-18 administered and/or present in the tumor microenvironment (67). For instance, low doses of IL-18 were shown to accelerate tumor progression (66), in part through the induction of Kit^+^ immunosuppressive NK cells, while daily administration of IL-18 induced inflammation and increased the anti-tumor response, thus suppressing tumor growth. The function of IL-18 in cancer could also depend on cytokines present at the site of NK cell activation and maturation. Recent studies have shown that incubation of NK cells with IL-18, IL-12, and IL-15 results in the generation of cytokine-induced memory-like (CIML) NK cells, which have enhanced cytokine (IFN-γ) production and exhibit potent anti-tumor effects against both hematological (76) and non-hematological malignancies (77). Studies aimed at establishing protocols for the *in vitro* generation of tumor-suppressive NK cells determined that radiation therapy greatly increased the number and function of the transferred NK cells, and was essential for the anti-tumor activity of transferred CIML NK cells (78). Thus, harnessing cytokine-induced memory-like NK cell responses could represent a promising translational immunotherapy approach for patients with malignant disease.

Given the studies demonstrating an anti-tumorigenic function for IL-18, numerous strategies are being investigated aimed at increasing the level of IL-18 in the tumor microenvironment and thus to augment the anti-tumor immune response. This includes the engineering of oncolytic adenoviruses co-expressing IL-18 and IL-12 (79). Intratumoral administration of these adenoviruses improved the anti-tumor immune response in the B16-F10 murine melanoma model, in part though the generation of an effective T-cell mediated immune response. Bacteria have also been used to deliver IL-18 into the tumor microenvironment to augment the T-cell mediated immune response (80). IL-18-producing attenuated *Salmonella typhimurium* inhibited the growth of primary subcutaneous tumors as well as pulmonary metastases in immunocompetent mice challenged with syngeneic multidrug-resistant clones of murine carcinoma cell lines, without overt toxicity to normal tissues.

## Interleukin-33

Initially identified as a nuclear factor, IL-33 was later determined to be a ligand for the receptor ST2 (Fit-1/IL-33R/ IL1RL1). A decoy receptor, sST2, has been found to be integral to the regulation and attenuation of the IL-33/ST2 axis, similar to that of IL1-R2 in interleukin-1 signaling (81, 82). IL-33 is expressed by a diverse range of cells, with the strongest expression observed in non-haematopoietic cells including endothelial cells, epithelial cells, keratinocytes, fibroblasts, fibrocytes and smooth muscle cells. Its receptor, ST2, is strongly expressed on the surface of fibroblasts and hematopoietic cells such as T helper type 2 (Th2) lymphocytes, ILC2s and mast cells. The IL-33/ST2 pathway has been investigated in many inflammatory diseases and dysregulation of this pathway has been particularly implicated in Th2-mediated conditions such as allergic diseases (83). The IL-33/ST2 signaling axis has been linked to processes involved in wound healing such as angiogenesis, production of matrix components, to fibrosis, and to modulation of immune populations (84). Given the critical importance of such pathways in tumor formation, links between the IL-33/ST2 signaling axis and tumorigenesis have recently been identified, with multiple research groups implicating IL-33 as a key player in the mediation of neoplastic transformation, tumor growth and metastasis in various cancers including breast, gastric, colorectal and lung (85). In parallel with other IL-1 family members, both pro- and anti-tumorigenic roles have been reported for IL-33 in cancer.

### Tumor Promoting Effects of IL-33

Several studies have examined expression of IL-33 and ST2, and their association with patient prognosis. IL-33 and ST2 are both elevated in comparison to adjacent healthy tissue in breast cancer, with serum levels of IL-33 and its decoy receptor soluble ST2 (sST2) also elevated. These expression changes also correlated with markers of poor prognosis (86). Similarly, expression of IL-33 was found to be increased when compared to adjacent healthy tissue in patients with both non-small-cell lung carcinoma (NSCLC) (87) and epithelial ovarian cancer (88). Maywald et al showed expression of IL-33 to be induced in colorectal cancer at both mRNA and protein levels in patient-derived cell lines, adenocarcinoma biopsies and in the systemic circulation of CRC patients as compared to adjacent non-tumor tissue (89).

Much of the early work examining IL-33 in tumorigenesis focussed on the role of IL-33 in breast cancer. Deletion of ST2 in BALB/c mice bearing mammary carcinoma attenuated tumor growth and metastasis (90). This was accompanied by increased serum levels of IL-17, IFN-γ and TNF-α, and decreased IL-4. Suppressing sST2 reduced ErbB2-induced cell motility in breast cancer cells (91). In a subsequent study (92), administration of exogenous IL-33 to breast cancer-bearing mice was seen to enhance tumor growth and metastasis. The mechanism proposed to be responsible for the enhanced tumor growth involves the ability of IL-33 to cause an increase in the number of infiltrating immunosuppressive immune cells and innate lymphoid cells to the tumors, drive the tumorigenic process. Subsequent *in vitro* and *in vivo* work on multiple cancer types have confirmed these findings, with a similar IL-33-induced immuno-suppressive microenvironment reported to play a tumor-promoting role in, amongst others, CRC, gastric cancer, lung cancer, head and neck cancers, pancreatic cancers and cervical cancer (93).

A role for IL-33 in the classic tumorigenic processes such as EMT, proliferation, migration and invasion, has now been extensively studied (84). Stromal CAFs are the main type of non-immune cells in the tumor microenvironment (TME), with CAFs known to interact with cancer cells to promote tumor proliferation. IL-33-producing CAFS have been implicated as a critical mediator in CAF-induced invasiveness in head and neck squamous cell carcinoma (HNSCC) (94). In this cancer type, administration of IL-33 promotes cancer cell migration and invasion through induction of EMT. In oral squamous cell carcinoma (OSCC), an interplay between a long non-coding (lnc)-RNA strongly upregulated in CAFs and IL-33 was recently established. IL-33 was found to be co-expressed with this lnc-CAF, resulting in elevation in the expression of CAF markers, conversion of the normal fibroblast phenotype to stromal carcinoma-related fibroblasts and enhanced tumor growth. This was achieved by the lnc-CAF promoting IL-33 stability (95). Intestinal epithelial cells, in turn, have been shown to use epidermal growth factor (EGF) as a key factor to modulate IL-33 production in cells. Inhibition of the EGF receptor resulted in lower levels of IL-33 transcripts, with increased EGF stimulation resulting in increased expression of ST2 and IL-33 (96). IL-33 has also been shown to promote tumor cell growth, colony formation and expression of Ki-67 and proliferating cell nuclear antigen (PCNA) in a PGE_2_ dependant manner (97).

Regarding tumor cell metastasis, *in vitro* assays have shown a direct ability of IL-33 to stimulate migration and invasion of the lung cancer cell line A549 (98). Moreover, Andersson and colleagues recently demonstrated that in mouse and human fibroblast-rich pancreatic cancers, genetic deletion of IL-33, ST2 or MMP-9 markedly blocked metastasis (99). This appeared to be due, in part, to modulation of the tumor microenvironment by IL-33-producing CAFs, which caused the transition of tumor-associated macrophages from an M1 (anti-tumorigenic) to an M2 (pro-tumorigenic) phenotype. Moreover, the decoy receptor sST2 has been found to have decreased expression in high-metastatic cells compared with low-metastatic human and mouse CRC cells (100). sST2 was found to negatively regulate tumor growth and the metastatic spread of CRC through modification of the tumor microenvironment. The expression of the IL-33 receptor, ST2L, has also been implicated in metastasis (101). Low-metastatic cells but not high-metastatic cells derived from Lewis lung carcinoma were found to express functional ST2L. IL-33 was seen to augment the cell death of ST2L-positive low-metastatic cells, but not of the ST2L-negative high-metastatic cells, suggesting that IL-33 enhances lung cancer progression by selecting for more malignant cells in the tumor microenvironment. Combined these data indicate the potential therapeutic benefit of blocking this pathway in tumorigenesis.

### Tumor-Inhibiting Effects of IL-33

Alongside the above listed pro-tumorigenic roles ascribed to the IL-33/ST2 pathway are several strands of evidence implicating a divergent role for IL-33 in the inhibition of tumorigenesis. Many reports focusing specifically on tumoral expression of IL-33 and ST2 have reported decreasing expression of these proteins with increasing tumor stage. In cervical intraepithelial neoplasia (CIN) tissues, IL-33 protein and mRNA levels in cervical tissues were significantly lower in severe CIN as compared to those patients with mild or no CIN (102). Levels of IL-33 have also been shown to negatively correlate with tumor stage in multiple myeloma patients (103) and lung cancer patients (104). Our laboratory also demonstrated that ST2L expression is decreased in human CRC tissue as compared to adjacent non-tumor tissue, with lower ST2L expression correlating with poorer patient prognosis (105). A similar pattern of expression has been demonstrated in metastases. IL-33 expression was shown to be reduced in many carcinomas upon their transition to the metastatic form of the disease (106). These authors described low tumoral expression of IL-33 as an immune biomarker associated with recurrent prostate and kidney renal clear cell carcinomas. Taken together these studies indicate that downregulation of the IL-33/ST2 axis in epithelial cells is tightly associated with tumorigenesis and metastasis, suggesting that retaining IL-33/ST2 expression in these cells might inhibit cancer progression.

A key theme emerging in the field of the anti-tumorigenic effects of IL-33 is the ability of Il-33 to induce Type 1 immune responses. Given the fact that the focus of IL-33/ST2 research was, for many years, related to its expression and involvement in type 2 immune cells and responses, this is something of a paradigm shift. Gao et al. investigated the effect of overexpressing IL-33 in both B16 melanoma cells and 4T1 breast cancer cells (107). Intradermal injection of these cells resulted in a greatly decreased tumor growth rate. Characterization of immune cells in the IL-33-expressing tumors revealed a local increase in CD8^+^ T cells and NK cells, with increased expression of type 1 effectors such as IFN-y, IL-12 and granzyme B. Moreover, IL-33 was shown to regulate local tumor-associated myeloid cells and enhance the expression of MHC II as a result of the type I immune response (108). Similarly, suppression of ST2 in colon cancer cells was seen to enhance tumor growth and reduce CD8^+^ T cell infiltration into the tumors (105).

Administration of exogenous IL-33 has also been shown to have potent tumor protective effects. Augmentation of the immune response by systemic administration of recombinant IL-33 was shown to inhibit leukemia growth and prolong the survival of leukemia bearing mice (109). This was mediated via an increase in CD8^+^ T cell and IFN-γ. Similarly, administration of recombinant IL-33 inhibited the growth of established melanoma tumors by activating myeloid dendritic cells and tumor associated CD8^+^ T cells (110). Exogenous administration of IL-33 has also been shown to limit cellular metastasis (111). Injection of IL-33 into mice-bearing subcutaneous B16-F10 melanoma cells resulted not only in significant attenuation of primary tumor growth but also in a reduction in pulmonary metastasis. Finally, introduction of IL-33 into metastatic tumors reduced the levels of circulating tumor cells and boosted immune recognition against metastatic tumors *in vivo* (106). Collectively, these data indicate that when high levels of IL-33 are administered, potent anti-tumor effects are observed.

Taken together, there can be no doubt that whilst IL-33 drives tumorigenesis in certain cancers and cell types much caution must be exerted surrounding future development of this cytokine as a therapeutic target.

## Interleukin-36

The IL-36 cytokines are a recently described subset of the IL-1 family of cytokines (112). The three members of this family, IL-36α, IL-36β, and IL-36γ, all share the same receptor complex, which is composed of the IL-36 receptor (IL36R/IL1RRP2/IL1RL1) and the IL1 Receptor accessory protein. A biological inhibitor to this complex has also been identified, the IL-36R antagonist (IL-36Ra). The IL-36 cytokines and their receptor are expressed by several tissues, particularly the lung, skin and intestine, as well as by immune cells such as monocytes, macrophages, dendritic cells and T cells (112, 113). Activity of the IL-36 cytokines relies on proteolytic cleavage, with, proteases released by neutrophils, in particular cathepsin G and elastase, recently identified shown to be potent activators of the IL-36 cytokines (114).

Similar to other IL-1 family members, IL-36 cytokines are important activators of the inflammatory response, stimulating both innate and adaptive immune responses (113). Whilst these cytokines have been shown to play an important role in autoimmune diseases, in particular in the pathogenesis of psoriasis and Inflammatory Bowel Diseases, few studies to date have investigated their role in cancer. Indeed, the majority of studies to date have focused on IL-36γ, with few, if any, investigating the role of IL-36α or IL-36β in cancer.

### Tumor-Inhibiting Effects of IL-36

One of the first studies identifying a role for IL-36γ in cancer determined that IL-36γ was anti-tumorigenic in breast cancer and melanoma (115). In particular, IL-36γ was shown to enhance the effector functions of CD8^+^ T cells, NK cells and γδ T cells, to transform the tumor microenvironment into one favoring tumor destruction, and ultimately to have profound anti-tumor effects, suppressing both tumor growth and metastasis. Chen et al. investigated this anti-tumorigenic role of IL-36γ (116). The group developed and showed a polymer-based delivery system that allowed the co-delivery of IL-36γ expression plasmid with doxorubicin (Dox). This showed that micelles loaded with IL-36γ and Dox significantly reduced the metastatic spread of breast cancer cells to the lung. This was associated with an enhanced type I immune response together with a reduction in the levels of immunosuppressive myeloid-derived suppressor cells in the lung. Moreover, injection of bioactive IL-36γ into the tumor microenvironment was also shown to delay tumor progression (117), via with the rapid recruitment of T cells and the formation of tertiary lymphoid organs (TLOs). TLOs form at sites of persistent inflammation and provide a specialized microenvironment for priming of naïve T cell into effector cells, with the presence of TLOs in a broad range of cancer types associated with improved clinical prognosis (118).

Subsequent studies in human colon cancer demonstrated the presence of IL-36γ in a variety of cell types within the tumor microenvironment, including immune cells, in particular in classically activated or M1 macrophages, tumor cells and vascular/perivascular cells (119). Interestingly, expression of the IL-36 receptor antagonist, IL-36RN, was associated with elevated expression of immune checkpoint molecules such as PD-1, PD-L1, and CTLA-4 which are well-characterized inhibitors of the anti-tumor immune response. Given that the binding affinity of IL-36γ to the IL-36R is ~ 100–1000-fold greater than IL-36RN, the ability of exogenously administered IL-36γ to augment the anti-tumor immune response and suppress tumor growth, may be, in part, due to a reversal in suppression mediated by these checkpoint inhibitors.

Of the remaining members of this family, IL-36α and IL-36β, to date, three studies in the literature have suggested that IL-36α may also have tumor-suppressive effects. IL-36α was shown to suppress the growth of ovarian cancer cells (120). Moreover, reduced expression of IL-36α has also been shown to be associated with poor prognosis in colon (121) and hepatocellular cancer (122). However, the mechanisms underlying this suppressive function are unknown. As such, the role of IL-36α or IL-36β in cancer remains poorly defined.

**Figure 2:**
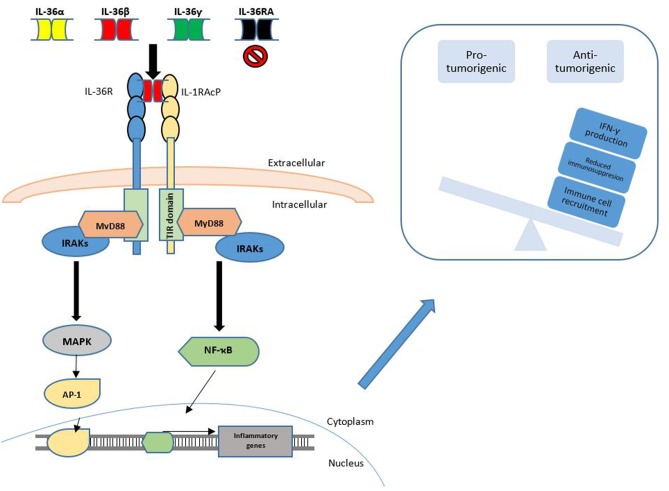
IL-36 Signaling in cancer. IL-36 signaling is achieved through binding of the IL-36α, IL-36β or IL-36γ cytokines to the IL-36 Receptor (IL1RL2), along with the transmembrane accessory protein, IL-1RAcP. This binding may be inhibited through competitive binding of IL-36RA or IL-38. Agonist binding is followed by subsequent engagement and phosphorylation of intracellular signaling molecules to activate transcription factors such as NF-κB and AP-1. This promotes the observed anti-tumorigenic phenotype, which may be achieved by increased IFN-γ production, reduced immunosuppression and increased immune cell recruitment to the local tumor microenvironment.

Interestingly, in contrast to other members of this family, to the best of our knowledge, no studies to date have determined that any of the IL-36 cytokines have tumor-promoting effects. Given the paucity of studies concerning IL-36α and IL-36β in tumorigenesis, further work is required to delineate the role of these family members in cancer and future studies may identify alternate tumor promoting effects of these cytokines. The known functions of IL-36 signaling in cancer are summarized in Figure 2.

## Interleukin-37

Similar to IL-1α and IL-33, IL-37 is a dual-function cytokine that can function as either a nuclear transcription factor or can activate signaling pathways by binding to its receptor on the cell surface (123, 124). The gene for IL-37 undergoes alternative splicing, resulting in the expression of five splice variants (IL-37a–e), although IL-37c and IL-37e are thought to encode non-functional proteins (123). IL-37b is the most abundant and most studied isoform, with almost all experimental published investigations focused on this isoform (124).

Expression of IL-37 is induced by pro-inflammatory stimuli, with IL-37 being synthesized as a precursor that is cleaved in the cytosol by caspase-1, generating mature IL-37. Both the precursor and mature forms are secreted by cells, and both forms are biologically active (124). Extracellular IL-37 binds to IL-18 receptor α (IL-18Rα; also used by IL-18), and recruits IL-1 receptor 8 (IL-1R8/TIR-8/SIGIRR) for the activation of intracellular signaling pathways, while the intracellular mature form of IL-37 binds to SMAD-3 in the cytoplasm, and then traffics to the nucleus. In contrast to the inflammatory function seen with other IL-1 family members, IL-37 is consistently an anti-inflammatory cytokine, with both extracellular and nuclear IL-37 functioning to suppress innate and adaptive immune responses by inhibiting the production of pro-inflammatory cytokines and chemokines (125, 126).

### Tumor-Promoting Effects of IL-37

While most studies investigating a role for IL-37 in cancer have determined that IL-37 is an anti-tumorigenic cytokine, some studies have suggested that IL-37 may also have pro-tumorigenic functions. Consistent with a role in tumor growth and metastasis, high levels of IL-37 in the serum of patients with ovarian cancer was associated with poor prognosis, poor overall survival and the progression-free survival (127). Similarly, IL-37 protein expression was shown to be increased during the malignant transformation of the oral mucosa, being elevated to precancerous lesions as well as in OSCC (128). However, IL-37 expression was lower in OSCC cells with lymph node metastasis than in those without metastasis, suggesting that IL-37 may play a role in cellular transformation, but once cells become malignant, IL-37 functions to inhibit the tumorigenic process.

Moreover, a recent study determined that IL-37 is a potent pro-angiogenic cytokine (129). IL-37 was shown to be upregulated by endothelial cells under hypoxic conditions, and to promote endothelial cell proliferation, capillary formation, migration and vessel sprouting from aortic rings. Given that tumor growth and metastasis depends on angiogenesis, the ability of IL-37 to promote the formation of new blood vessels in hypoxia regions of tumors could represent an important pro-tumorigenic function for IL-37. However, whether IL-37 functions as a pro-angiogenic factor in tumors is currently unknown.

### Tumor-Inhibiting Effects of IL-37

Given its ability to suppress the production of inflammatory factors, it is unsurprising that the majority of studies investigating the role of IL-37 in cancer have shown that IL-37 is decreased in cancer, with low levels associated with poor prognosis (130–133). Moreover, studies have determined that IL-37 can suppress tumor progression, migration, invasion and metastasis in multiple cancer types, including fibrosarcoma (134), hepatocellular carcinoma (HCC)(135), cervical cancer (136), lung cancer (137) and colon cancer (138). Consistent with these studies, intratumoral injection of IL-37 or over-expression of IL-37 by tumor cells inhibited tumor growth (130, 132, 134). Several mechanisms have been proposed, including suppression of STAT3 signaling (130, 136, 137). Activated STAT3 is known to increased cell proliferation and invasion, and to promote oncogenic inflammation, with IL-37 shown to inhibit both the expression and phosphorylation of STAT3. IL-37 has also been shown to inhibit epithelial-mesenchymal transition (137, 139, 140), which plays an important role in metastasis. Additional mechanisms proposed for the anti-metastatic function include inhibition of Rac1 activation (133), with the intracellular mature form of IL-37, but not its extracellular form, markedly inhibiting the migration of multiple tumor types.

IL-37 has also been shown to affect immune cell recruitment to the tumor microenvironment (132). IL-37 expression positively correlated with the density of tumor infiltrating CD57^+^ NK cells in HCC tumors, which were significantly associated with better overall survival in HCC patients. Moreover, overexpression of IL-37 by hepatic tumor cells resulted in enhanced local CD57^+^ NK cell infiltration of the tumors and suppressed tumor growth *in vivo* (132). This relationship between high intratumoral levels of NK cells and increased survival has been shown in several cancer types (141). Moreover, given that IL-37 suppresses inflammatory cytokine production, IL-37 could potentially alter the tumor microenvironment in favor of one that promotes tumor rejection. For instance, IL-37 reduces the secretion by both immune cells (142) and tumor cells (133) of IL-6, IL-1β and tumor necrosis factor alpha, cytokines known to play a role in tumor progression.

Finally, consistent with IL-37b being the most abundant isoform of IL-37, studies investigating tumor expression of the IL-37 splice variants identified IL-37b as the isoform expressed by breast, liver, lung and prostate cancer, with low levels of IL-37b significantly associated with poor prognosis (130). Whether this variant of IL-37 is important in suppressing tumor growth and metastasis in all forms of cancer is unknown.

Taken together, the major function of this member of the IL-1 family is to suppress the development and growth of tumor cells, and suggest that it represents a novel target for the treatment of neoplastic disease.

## Interleukin-38

IL-38 is the most recent member of the IL-1 family to be identified. It was discovered by two independent research groups in 2001 (143, 144). IL-38 shares structural features with both IL-1Ra and IL-36Ra, suggesting that it might act as an IL-1 family antagonist. Indeed, one of the first described biological functions of IL-38 was blocking the activation of the IL-36R, similar to that of IL-36Ra (145). In addition to binding to the IL-36R, studies have also proposed that IL-38 can bind to IL-1R1, or can interact with one of the inhibitory co-receptors of the IL-1R family, namely SIGIRR, TIGIRR1 and/or TIGIRR2 (146, 147). Similar to other family members, IL-38 is also processed by proteolytic cleavage, although there is currently a lack of consensus on the cleavage site.

IL-38 is expressed in several tissues including tonsils, placenta, heart and brain, and has been implicated in a variety of diseases including cardiovascular and autoimmune disease. In general, IL-38 polymorphisms are associated with increased susceptibility for auto-inflammatory diseases such as spondyloarthritis, rheumatoid arthritis, and psoriatic arthritis, suggesting a role of IL-38 suppressing chronic inflammation (147). Consistent with this, overexpression of IL-38 attenuated the severity of experimental arthritis, in part by decreasing the production of pro-inflammatory cytokines by macrophages and synovial fibroblasts (148).

### Tumor-Promoting Effects of IL-38

Few studies to date have investigated the role of IL-38 in cancer. One of the first studies investigating expression of IL-38 in cancer demonstrated that tumor expression of IL-38 was increased in multiple cancer types, and that high expression of IL-38 was associated with poor prognosis in lung adenocarcinoma (149).

### Tumor-Inhibiting Effects of IL-38

In contrast, Wang et al. demonstrated that IL-38 expression is decreased in NSCLC alone relative to adjacent non-tumor tissue (150), with lack of IL-38 expression correlating with poor prognosis. Administration of recombinant IL-38 inhibited β-catenin expression and reduced the proliferation, migration and invasion of lung cancer cells *in vitro*, while increasing cell death. Consistent with a role for IL-38 in suppressing lung cancer, overexpression of IL-38 suppressed NSCLC development *in vivo* and increased the sensitivity to chemotherapeutic drugs. The reason for these divergent results is unclear, but may be due to differential processing of the precursor form of IL-38 by the different tumor types. Studies comparing the function of full-length IL-38 vs. truncated IL-38 in macrophages (151) demonstrated that high concentrations of the truncated form decreased IL-1β-induced IL-6 production in macrophages. However, the full-length form of IL-38 increased IL-6 production at these concentrations, suggesting the biological function of IL-38 is affected by processing *in vivo*. Although IL-38 is well characterized as an anti-inflammatory cytokine (147), the role of IL-38 in cancer remains to be determined.

## Conclusions

The IL-1 superfamily is a diverse family of cytokines, most of whose members have been shown to have dual functions in tumorigenesis with both pro- and anti-tumorigenic roles being ascribed (Table 1). The role of these cytokines varies greatly depending on the tissues and organs involved, the inflammatory background and the stage of the cancer. An additional factor determining the pro- or anti- tumorigenic effects of these cytokines appears to be whether the cytokines are produced by the tumor-infiltrating immune populations or by the tumor cells. Finally, it is possible that the levels of the cytokines present within the tumor microenvironment may have a role in determining the outcome of the effect on tumorigenesis. For example, when the pro-inflammatory cytokines are produced at persistent, chronic low levels, often by the tumor itself, this could play a role in shaping an immune-suppressive tumor microenvironment, facilitating a chronic inflammatory state and enabling tumor growth and spread. In line with this, results from the CANTOS trial imply that suppressing such chronic inflammatory effects of these cytokines over an extended period may have significant benefits in terms of limiting cancer development and progression. In contrast, exogenous administration of high levels of these cytokines has been shown to often have a potent anti-tumorigenic effect due to recruitment and activation of Type 1 immune responses. Further work is required in this field to determine the levels of these cytokines within both normal and tumor tissue.

**Table 1:** Pro- and anti-tumorigenic functions of IL-1 family members in cancer.

	**Function**	**Mechanism**	**Tumor Type**
IL-1α	Pro-tumorigenic	Angiogenesis Cell growth Dedifferentiation Invasion & Metastasis Proliferation Tumor-promoting inflammation	Pancreatic cancer (19), Lung cancer (14) ALL (16) Breast cancer (13) HNSCC (11), Gastric Cancer (12), Pancreatic cancer (17, 19), Breast cancer (21) Pancreatic cancer (19), Breast Cancer (21, 22) Pancreatic cancer (17), Fibrosarcoma (18)
	Anti-tumorigenic	Immune cell recruitment Proliferation	Fibrosarcoma (25), lymphoma (26) Breast Cancer (27), Melanom, Prostate Cancer (28)
IL-1β	Pro-tumorigenic	Angiogenesis Cell growth Invasion & Metastasis Proliferation TME modulation	Fibrosarcoma (32), Lung cancer (34), Pancreatic cancer (35), Melanoma (36), Breast cancer (38) Fibrosarcoma (32) Breast cancer (37–39), Lung cancer (45), CRC (50) Breast cancer (38), Fibrosarcoma (32) Breast cancer (37)
	Anti-tumorigenic	Immune cell recruitment Metastasis	Fibrosarcoma (46), lymphoma (48), B-cell myeloma (47) Breast cancer (51)
IL-18	Pro-tumorigenic	Angiogenesis Invasion & Metastasis Immune evasion Migration	Gastric cancer (58) Melanoma (61, 62), HCC (55) Melanoma (64, 65, 67), Colon Carcinoma (64, 65) Myeloma (66) HCC (61), Breast cancer (63)
	Anti-tumorigenic	Immune cell modulation	Melanoma (70, 71, 78), CRC7 (3), Lung cancer (74), Myeloid leukaemia (75), Ovarian Cancerm (76), Breast Cancer (79)
IL-33	Pro-tumorigenic	Cell growth Immune evasion Invasion & Metastasis Migration TME modulation	OSCC (96), Colon cancer (97), CRC (98, 101) Breast Cancer (91), HNSCC (95), Pancreatic Cancer (92), Cervical Cancer (92) Lung Cancer (86, 99, 102), Breast cancer (89–91), Ovarian Cancer (87), CRC (88, 101) HNSCC (95), Lung Cancer (99) CRC (88, 93, 94), Gastric Cancer (92), Lung Cancer (94), Pancreatic Cancer (92)
	Anti-tumorigenic	Immune modulation Metastasis	Cervical Cancer (103) Multiple Myeloma (104), Lung Cancer (105), CRC (106), Melanoma (108, 111), Breast Cancer (108) Prostate Cancer (107), Renal Clear Cell Carcinoma (107)
IL-36	Pro-tumorigenic	N/A	N/A
	Anti-tumorigenic	Metastasis TME modulation	Breast Cancer (117) Breast Cancer (116, 117), Melanoma (116), Fibrosarcoma (118), Colon cancer (118, 120)
IL-37	Pro-tumorigenic	Cell transformation Metastasis	OSCC (129) Ovarian Cancer (128)
	Anti-tumorigenic	TME modulation Proliferation Invasion & Metastasis Migration	Renal Cell Carcinoma (132), HCC (133, 136), Fibrosarcoma (135), Colon Cancer (139) Cervical Cancer (137), Lung Cancer (140), Cervical Cancer (131, 137), Lung cancer (131, 134, 138), Gallbladder Cancer (141), Breast Cancer (131), Liver adenocarcinoma (131), Prostate Cancer (131) Lung Cancer (140), Gallbladder cancer (141), OSCC (123)
IL-38	Pro-tumorigenic	TME modulation	Lung Cancer (150)
	Anti-tumorigenic	Invasion Migration Proliferation	Lung Cancer (151) Lung Cancer (151) Lung Cancer (151)

There is clear potential for the development of agents to target these cytokines, whose administration, most likely as a combination therapy with checkpoint inhibitor proteins, will activate the anti-tumor immune response and lead to tumor regression. Given the complexity and the dual functions associated with these proteins, the development of these cytokines as therapeutic agents will require extensive knowledge and understanding of the specific functions of each member of this family in each individual cancer type. Finally further work regarding the more novel members of this family is required to firmly establish their role in tumor development and progression.

## Author Contributions

All authors listed have made a substantial, direct and intellectual contribution to the work, and approved it for publication.

### Conflict of Interest Statement

The authors declare that the research was conducted in the absence of any commercial or financial relationships that could be construed as a potential conflict of interest.
